# CKD prevalence based on real-world data: continuous age-dependent lower reference limits of eGFR with CKD–EPI, FAS and EKFC algorithms

**DOI:** 10.1007/s11255-022-03210-8

**Published:** 2022-04-28

**Authors:** Jakob Adler, Elina Taneva, Thomas Ansorge, Peter R. Mertens

**Affiliations:** 1 Medical Laboratory for Clinical Chemistry, Microbiology, Infectious Diseases and Genetics “Prof. Schenk/Dr. Ansorge & Colleagues”, Schwiesaustr. 11, 39124 Magdeburg, Germany; 2 Clinic of Nephrology and Hypertension, Diabetes and Endocrinology, Otto-Von-Guericke-University Magdeburg, Leipziger Straße 44, 39120 Magdeburg, Germany

**Keywords:** eGFR, CKD, Chronicity criterion, Age-dependent lower reference limits, Prevalence of CKD

## Abstract

**Purpose:**

Several recent articles discuss the need for a definition of chronic kidney disease (CKD) that embarks age-dependency and its impact on the prevalence of CKD. The relevance is derived from the common knowledge that renal function declines with age. The aim of this study was to calculate age-dependent eGFR lower reference limits and to consider their impact on the prevalence of CKD.

**Methods:**

A real-world data set from patients with inconspicuous urinalysis was used to establish two quantile regression models which were used to calculate continuous age-dependent lower reference limits of CKD–EPI, FAS and EKFC–eGFR based on either single eGFR determinations or eGFR values that are stable over a period of at least 3 months (± 10% eGFR). The derived lower reference limits were used to calculate the prevalence of CKD in a validation data set. Prevalence calculation was done once without and once with application of the chronicity criterion.

**Results:**

Both models yielded age-dependent lower reference limits of eGFR that are comparable to previously published data. The model using patients with stable eGFR resulted in higher eGFR reference limits. By applying the chronicity criterion, a lower prevalence of CKD was calculated when compared to one-time eGFR measurements (CKD–EPI: 9.8% vs. 8.3%, FAS: 8.0% vs. 7.2%, EKFC: 9.0% vs. 7.1%).

**Conclusion:**

The application of age-dependent lower reference intervals of eGFR together with the chronicity criterion result in a lower prevalence of CKD which supports the estimates of recently published work and the idea of introducing age-dependency into the definition of CKD.

## Introduction

In recent years, ongoing discussions on the need for an age-dependent definition of chronic kidney disease (CKD) have been ignited [1]. It is consensus that renal function declines with age, yet it has not been delineated whether age-related deterioration in renal function is a phenomenon predominantly linked with cell senescence and loss of nephron number versus occurrence due to underlying diseases that are more prevalent with advanced age [2]. Yet, the CKD definition in Kidney Disease—Improving Global Outcomes (KDIGO) guideline lacks an age-dependency hitherto [3]. To address this issue, Delanaye et al. proposed an age-dependent three-stage model in which “normal range cut-offs” for estimated glomerular filtration rates (eGFR) are defined to separate impaired renal function in dependency of age (75 ml/min/1.73 m^2^ (< 40 years), 60 ml/min/1.73 m^2^ (40–65 years), 45 ml/min/1.73 m^2^ (> 65 years) [4]. This three-stage model is a simplification of their continuous model, which uses the 3rd percentile as a lower limit [4–6]. It is important to point out that reference intervals and clinical decision limits are mostly not congruent. While a reference interval is a statistically determined range between the 2.5th and the 97.5th percentiles of the measured values of a biomarker in an “apparently healthy” reference cohort, consisting of a lower and upper reference limit, clinical decision limits are determined from clinical studies dealing with specific diseases [7]. The current definition of CKD is based on clinical decision limits. Since it is known that eGFR decreases steadily beyond the age of about 40 years even in healthy individuals [4], the application of a definition that assumes CKD at an eGFR below 60 ml/min even in the absence of renal pathology, such as proteinuria or pathologic imaging, is likely to result in an over-diagnosis of CKD, as discussed by Glassock et al. [1]. In addition, excess mortality of people older than 65 years with an eGFR ranging from 45 to 59 ml/min does not appear to be significantly higher than that of people of the same age with an eGFR ranging between 75 and 89 ml/min [4, 8]. These data indicate that the definition of CKD should be age-dependent to avoid over-diagnosis of CKD [1].

With age-dependent definition of CKD, the lower reference limits of eGFR not only have an impact on the diagnosis of impaired kidney function for each individual, but it also furthermore skews the overall prevalence rates of CKD. Another important influence on CKD prevalence is the presence or absence of chronicity of renal impairment. Many studies relied on one-time eGFR measurements and failed to meet the chronicity criterion put forward by the KDIGO definition of CKD with respect to impaired renal function and/or persistence of proteinuria [9]. The derived estimates conclude that the prevalence of CKD ranges between 10 and 16% [9]. However, recent studies suggest that the prevalence of CKD is lower following strict adherence to KDIGO criteria together with applying age-dependent eGFR thresholds and calculates numbers around 6% [9, 10, 16].

In this study, we establish two models for age-dependent lower reference limits of Chronic Kidney Disease Epidemiology Collaboration (CKD–EPI)–eGFR [11], Full Age Spectrum (FAS)-eGFR [12] and European Kidney Function Consortium (EKFC)-eGFR [13], that are based on one-time eGFR determinations and eGFR values that are stable over a period of at least 3 months, respectively. The data are derived from a real-world data set as outlined in the data collection and modeling section. The two models are used to predict continuous age-dependent lower reference limits of eGFR for all three eGFR formulas. The resulting lower reference limits are applied to calculate the prevalence of CKD in a validation data set, once without and once with appliance of the chronicity criterion, to obtain insights into the impact of the chronicity criterion for the calculated prevalence rates of CKD.

## Data collection and modeling

**Fig. 1:**
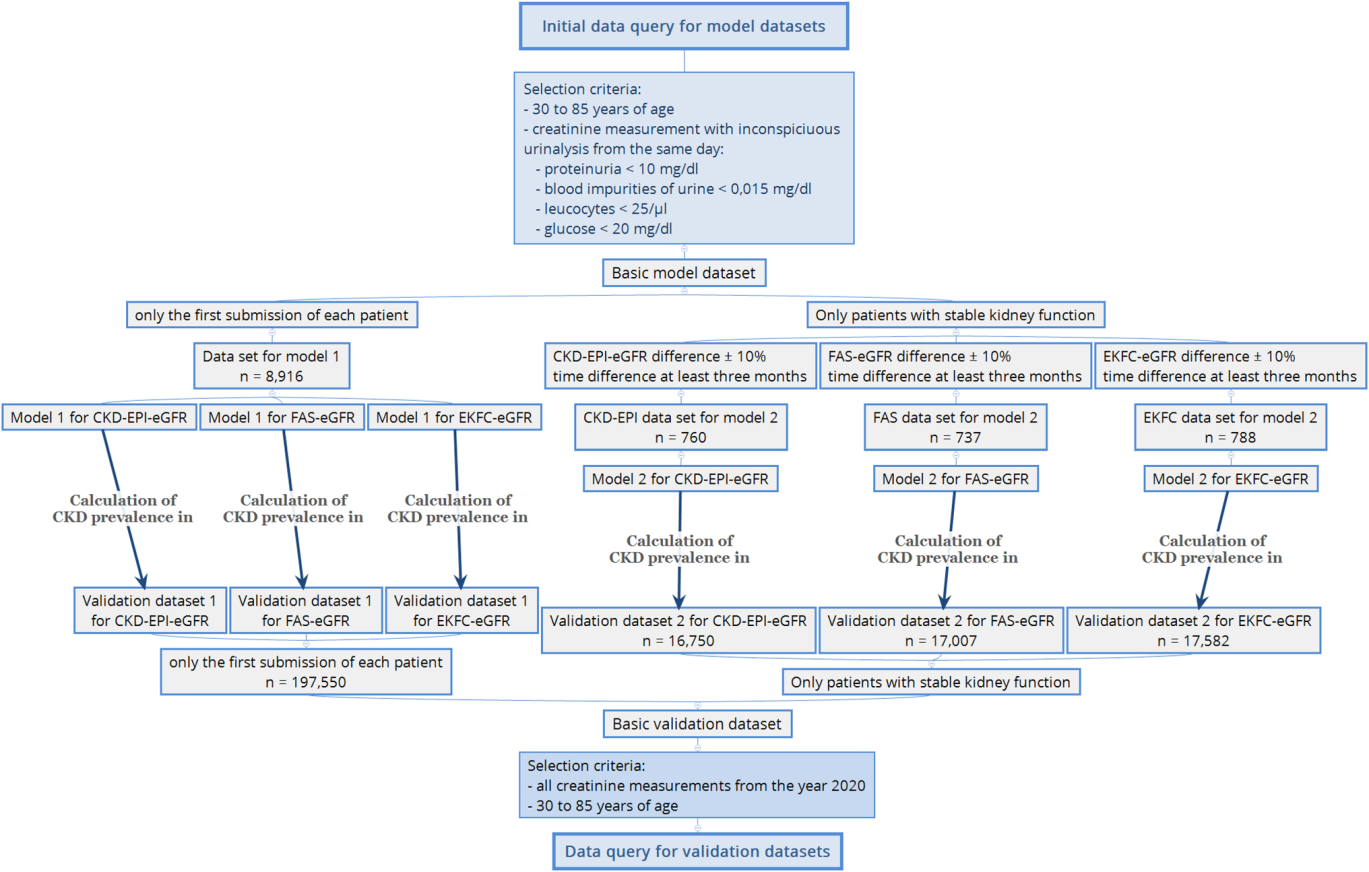
Overview of workflow for creating the data sets and calculating the models

For a better understanding, the workflow for creating the data sets and calculating the models and the prevalence of CKD is visualized in Fig. 1. The characteristics of the data sets and models are provided in Table 1.

**Table 1:** Characteristics of the data sets and models

Criterion	Model data sets				Validation data sets			
Data set	Data set for model 1	Data set for model 2—CKD–EPI–eGFR	Data set for model 2—FAS-eGFR	Data set for model 2—EKFC-eGFR	Validation data set 1	Validation data set 2—CKD–EPI–eGFR	Validation data set 2—FAS-eGFR	Validation data set 2—EKFC-eGFR
n (included patients)	8,916	760	737	788	197,550	16,750	17,007	17,582
Age (range; years)	30.0–85.0				30.0–85.0			
Age (mean (SD); years)	57.4 (14.1)	58.5 (15.6)	58.4 (15.8)	58.5 (15.6)	63.0 (13.9)	65.5 (12.7)	65.9 (12.6)	65.5 (12.7)
CKD–EPI–eGFR (range; ml/min)	11.6–148.4	20.7–119.7	–	–	2.1–174.5	8.5–149.2	–	–
CKD–EPI–eGFR (mean (SD); ml/min)	82.5 (17.6)	83.3 (17.4)	–	–	75.0 (21.2)	74.4 (20.1)	–	–
FAS–eGFR (range; ml/min)	14.1–204.4	–	22.0–152.6	–	3.3–477.8	–	9.8–259.1	–
FAS–eGFR (mean (SD); ml/min)	79.2 (20.0)	–	77.4 (19.5)	–	71.0 (22.6)	–	66.8 (20.2)	–
EKFC–eGFR (range; ml/min)	12.0–132.0	–	–	20.4–113.1	2.4–167.6	–	–	7.4–135.2
EKFC–eGFR (mean (SD); ml/min)	78.8 (17.7)	–	–	79.1 (17.4)	70.9 (20.6)	–	–	69.8 (19.4)
Kidney function stable for at least 3 months (eGFR deviation maximum ± 10%)	No	Yes	Yes	Yes	No	Yes	Yes	Yes
Time difference between measurements (range; years)	–	0.3–3.0	0.3–3.0	0.3–3.0	-	0.25–0.98	0.25–0.98	0.25–0.98
Time difference between measurements (mean (SD); years)	–	1.49 (0.71)	1.49 (0.71)	1.50 (0.71)	-	0.49 (0.15)	0.49 (0.15)	0.49 (0.15)
eGFR difference between measurements (range; %)	–	– 9.99 to 9.96	− 9.9 to 9.97	− 9.98 to 9.95	–	− 9.99 to 9.99	− 10.0 to 10.0	− 9.99 to 10.0
eGFR difference between measurements (mean (SD); %)	–	0.62 (5.17)	0.79 (5.26)	0.75 (5.01)	–	1.13 (5.18)	1.21 (5.36)	1.20 (5.08)
Leukocyturia excluded	Yes				No			
Glucosuria excluded	Yes				No			
Erythrocyturia excluded	Yes				No			
Proteinuria excluded	Yes				No			
Patients from Nephrology practices and Dialysis centers included	No				Yes			
Regression method for continuous lower reference limits	2.5th percentile quantile regression with cubic splines and one knot at 40 years				Only for validation			

Comparison of the different data sets for the modeling and the validation as well as the methodology for calculating the models

In this study, we used a retrospective approach. All data were determined and collected at a regional privately organized laboratory specialized in providing blood testing for private practitioners (approximately 800 private practices and 8 hospitals) in an urban setting of a medium-sized city in central Germany (~ 250 k inhabitants).

### Model data sets

The basic model data set was created by a data query from the laboratory information system (data query period: August 2017–December 2020) from patients aged 30–85 years with a enzymatic quantification of creatinine (“Creatinine plus ver.2” (CREP2), Roche Diagnostics GmbH, Mannheim, Germany) as well as proteinuria < 10.0 mg/dl, blood impurities of urine < 0.015 mg/dl, < 25 leukocytes per µl of urine and < 20.0 mg/dl of glucose in the urine (measured using the iChemVELOCITY automated urinalysis chemistry instrument by Beckman Coulter GmbH, Krefeld, Germany). Blood and urine samples have been sent together to the laboratory and analyzed the same day. For each enzymatic creatinine measurement, the CKD–EPI, FAS and EKFC–eGFR was calculated. Submissions from hospitals, nephrology practices and dialysis centers were excluded. With this source data, the following data sets were created:

*Data set for model 1* Only the first submission of each patient into the query period was extracted, resulting in a data set of 8916 singular creatinine measurements with their corresponding eGFR calculations. This data set was used to calculate model 1 for CKD–EPI, FAS and EKFC–eGFR. The characteristics of the data set are provided in Table 1.

*Data sets for model 2* Only patients with stable kidney function were included to exclude patients with acute kidney injury despite unremarkable urinalysis. According to the definition of CKD, which requires impaired kidney function for at least 3 months, stable kidney function was defined as an eGFR difference of less than ± 10% over a period of at least 3 months. Among the basic model data set, all patients with two or more submissions of specimens into the query period were selected. The first and the last measured value of each of these patients were extracted. Of these, only those were selected that showed a minimum time interval of 3 months between both measurements and a maximum change in eGFR of ± 10%. Based on the calculation of eGFR using three different formulas, three data sets result, each containing the patients with stable kidney function for each eGFR formula. The characteristics of these three data sets are summarized in Table 1.

### Modeling

As outlined, the lower reference limit of a reference interval is defined as the 2.5th percentile [7]. To calculate continuous age-dependent lower reference limits of eGFR based on the 2.5th percentile, first, the age of the patients was rounded down. For the data set for model 2, the age of the patient at the time of the later eGFR measurement was selected to separate the patients into annual age groups from 30 to 85 years. In the following, a quantile regression model was calculated from the data set for model 1 for each eGFR formula and for all three data sets for model 2 for each eGFR formula (the later eGFR measurement was selected), resulting in six models. Other variables such as gender were not adjusted in the models. The same meta-parameters were used for all models. The regression was based on the 2.5th percentile. Cubic splines were used to smooth the curve. Due to the known physiological decrease of kidney function from an age of approximately 40 years [4], a knot was inserted at 40 years of age. After the models were created, the CKD–EPI, FAS and EKFC–eGFR age-dependent continuous lower reference limits were predicted. An excerpt of these results is shown in Table 2.

**Table 2:** Sample extract of the predicted eGFR lower reference limits (2.5th percentile) at 5-year intervals from 30 to 85 years

Age (years)	Model 1 CKD–EPI–eGFR (ml/min)	Model 1 FAS-eGFR (ml/min)	Model 1 EKFC-eGFR (ml/min)	Delanaye model [4] FAS-eGFR (ml/min)	Model 2 CKD–EPI–eGFR (ml/min)	Model 2 FAS-eGFR (ml/min)	Model 2 EKFC-eGFR (ml/min)
30	76	74	72	81^a^	84	84	82
35	72	75	71	81^a^	74	78	75
40	69	73	69	81	71	74	71
45	66	69	66	76	69	70	68
50	63	64	63	72	65	65	64
55	59	59	58	67	61	59	60
60	54	54	53	63	57	53	55
65	49	47	47	60	52	47	51
70	43	41	41	56	47	42	45
75	36	35	34	53	42	37	40
80	29	28	28	50	37	33	35
85	22	22	21	47	32	30	30

Model 1: quantile regression model without application of the chronicity criterion (2.5th percentile); Model 2: quantile regression model with application of the chronicity criterion (2.5th percentile); Delanaye´s continuous model (2.5th percentile) calculated from [4]

^a^In Delanaye’s conitnuous model, there is no factor for the age-dependent lowering of the lower reference limit for patients under 40 years of age

### Validation data sets

To estimate the prevalence of CKD based on the created models, we used a second basic data set. For the creation of this basic validation data set, all creatinine measurements submitted to the laboratory from 01.01.2020 to 31.12.2020 were queried, resulting in a data set of 443,548 creatinine measurements.

*Validation data set 1* To build a validation data set without appliance of the chronicity criterion, we extracted only the first value of each patient, resulting in a data set of 197,550 creatinine measurements. For each creatinine measurement, the CKD–EPI, FAS and EKFC–eGFR were calculated. The characteristics of these validation data sets are summarized in Table 1.

*Validation data set 2* To build a validation data set with appliance of the chronicity criterion, we extracted only patients from whom two measurements were available. Like model 2, only those were selected that showed a minimum time interval of 3 months between both measurements and a maximum change in CKD–EPI, FAS, or EKFC–eGFR of ± 10%. Based on the calculation of eGFR using three different formulas, three versions of validation data set 2 results, each containing the patients with stable kidney function for each eGFR formula. The characteristics of these three validation data sets are provided in Table 1.

To calculate the CKD prevalence, the numbers of all patients who were below the calculated lower reference limits in their age group were summed up and divided by the total number of patients in the respective validation data set as outlined in Fig. 1.

### Statistical software

Statistical analysis was performed using R statistic programming language (R Core Team (2021. R: A language and environment for statistical computing. R Foundation for Statistical Computing, Vienna, Austria, URL: https://www.R-project.org/.). For calculation of quantile regression models, the additional software packages “quantreg” and “splines” were used [14, 15]. Calculation of confidence intervals was done using the normal approximation.

### Approval of the local Ethics Committee

The local ethics committee of the Land Saxony-Anhalt ruled that a consent from the patients from whom the specimens originated were not required, due to the retrospective and fully anonymized setting of data evaluation (approval No. 17/21).

## Results

**Fig. 2:**
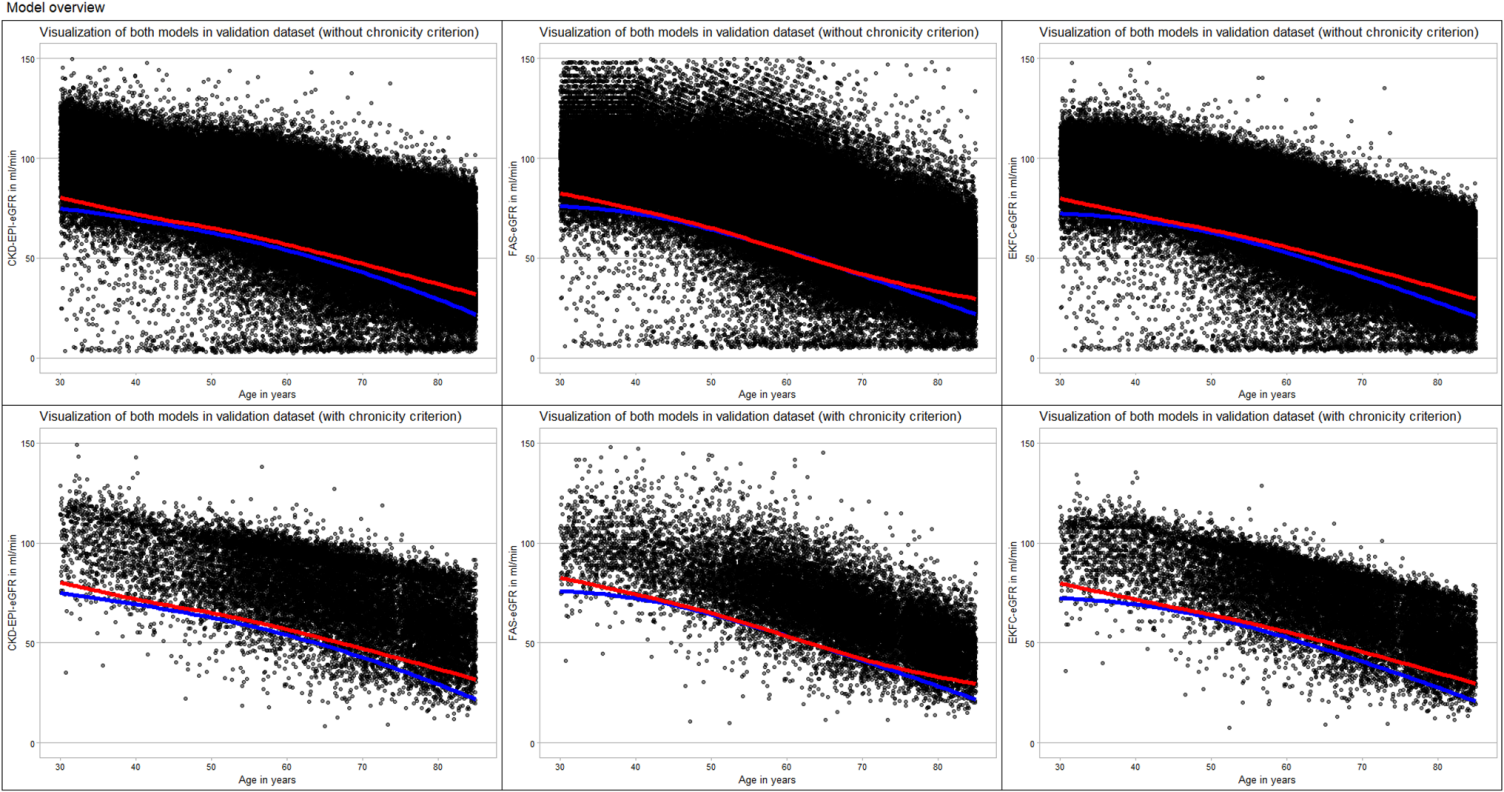
Visualization of the two models projected onto the validation data sets. Representation of the two models with lower reference limits indicated in the scatterplot of the validation data set. Model 1 (without chronicity criterion) is shown as blue line and model 2 (with chronicity criterion) is shown as red line

This study reveals two main findings:

(i) Using eGFR estimates originating from patients with stable renal function (eGFR difference at most ± 10% within a period of at least 3 months), higher continuous age-dependent reference limits are obtained than with one-time eGFR estimates entered into modeling. An extract of the predicted continuous age-dependent lower reference limits of eGFR in 5-year increments is provided in Table 2. In model 1 (2.5th percentile, inconspicuous urinalysis, one-time eGFR measurements), CKD–EPI–eGFR decreased from 76 to 22 ml/min, FAS–eGFR from 74 to 22 ml/min and EKFC–eGFR from 72 to 21 ml/min as a function of age. In model 2 (2.5th percentile, inconspicuous urinalysis, stable kidney function), CKD–EPI–eGFR decreased from 84 to 32 ml/min, FAS–eGFR from 84 to 30 ml/min and EKFC–eGFR from 82 to 30 ml/min as a function of age. For both models, these results also show the comparability of the three different eGFR estimates. The visualization of the two models for each eGFR formula together with the validation data sets is provided in Fig. 2.

(ii) Applying the chronicity criterion results in a lower prevalence of CKD, as previous work has already indicated [9, 10]. Using model 1 (2.5th percentile, inconspicuous urinalysis, single eGFR measurements) the CKD prevalence decreased from 6.4% (95%-CI 6.3–6.5) to 4.8% (95%-CI 4.5–5.1) for CKD–EPI–eGFR, from 6.3% (95%-CI 6.2–6.4) to 5.4% (95%-CI 5.1–5.7) for FAS–eGFR and from 7.3% (95%-CI 7.2–7.4) to 5.4% (95%-CI 5.1—5.7) for EKFC-eGFR when the chronicity criterion is applied.

Using model 2 (2.5th percentile, inconspicuous urinalysis, stable kidney function) higher prevalence rates were estimated but the effect of a lower prevalence was maintained when the chronicity criterion was applied. The prevalence of CKD decreases from 9.8% (95%-CI 9.7–9.9) to 8.3% (95%-CI 7.9–8.7) for CKD–EPI–eGFR, from 8.0% (95%-CI 7.9–8.1) to 7.2% (95%-CI 6.8–7.6) for FAS–eGFR and from 9.0% (95%-CI 8.9–9.1) to 7.1% (95%-CI 6.7–7.5) for EKFC–eGFR. Calculated prevalence rates independent of age are summarized in Table 3. Prevalence rates depending on age groups according to the three-stage model of Delanaye et al. (< 40 years, 40–65 years, > 60 years) [4] are shown in Table 4.

**Table 3:** Comparison of calculated prevalence rates between the two models, one without and one with appliance of the chronicity criterion

eGFR formula	Model	Validation data set 1 without chronicity criterion, CKD prevalence in % (95%-CI)	Validation data set 2 with chronicity criterion, CKD prevalence in % (95%-CI)
CKD–EPI	Model 1	6.4 (6.3–6.5)	4.8 (4.5–5.1)
	Model 2	9.8 (9.7–9.9)	8.3 (7.9–8.7)
FAS	Model 1	6.3 (6.2–6.4)	5.4 (5.1–5.7)
	Model 2	8.0 (7.9–8.1)	7.2 (6.8–7.6)
EKFC	Model 1	7.3 (7.2–7.4)	5.4 (5.1–5.7)
	Model 2	9.0 (8.9–9.1)	7.1 (6.7–7.5)

Model 1: quantile regression model without application of the chronicity criterion (2.5th percentile); Model 2: quantile regression model with application of the chronicity criterion (2.5th percentile)

**Table 4:** Prevalence of CKD depending on the age group and the selected model and validation data set

eGFR formula	Model	Age group in years	*n* in Validation data set 1	Validation data set 1 without chronicity criterion, CKD prevalence in % (95%-CI)	*n* in Validation data set 2	Validation data set 2 with chronicity criterion, CKD prevalence in % (95%-CI)
CKD–EPI	Model 1	30–40	17,802	4.7 (4.4–5.0)	3295	1.1 (0.8–1.4)
		41–65	90,104	6.2 (6.0–6.4)	7475	4.6 (4.1–5.1)
		66–85	89,644	7.0 (6.8–7.2)	5980	7.1 (6.4–7.8)
	Model 2	30–40	17,802	7.6 (7.2–8.0)	3295	1.6 (1.2–2.0)
		41–65	90,104	8.0 (7.8–8.2)	7475	6.3 (5.7–6.9)
		66–85	89,644	12.0 (11.8–12.2)	5980	14.4 (13.5–15.3)
FAS	Model 1	30–40	17,802	4.7 (4.4–5.0)	3344	1.3 (0.8–1.6)
		41–65	90,104	6.1 (5.9–6.3)	7600	5.3 (4.8–5.8)
		66–85	89,644	6.9 (6.7–7.1)	6063	7.8 (7.1–8.5)
	Model 2	30–40	17,802	7.8 (7.4–8.2)	3344	2.0 (1.5–2.5)
		41–65	90,104	6.3 (6.1–6.5)	7600	5.4 (4.9–5.9)
		66–85	89,644	9.7 (9.5–9.9)	6063	12.3 (11.5–13.1)
EKFC	Model 1	30–40	17,802	7.2 (6.8–7.6)	3454	1.7 (1.3–2.1)
		41–65	90,104	7.1 (6.9–7.3)	7850	5.4 (4.9–5.9)
		66–85	89,644	7.5 (7.3–7.7)	6278	7.6 (6.9–8.3)
	Model 2	30–40	17,802	11.5 (11.0–12.0)	3454	2.7 (2.2–3.2)
		41–65	90,104	7.4 (7.2–7.6)	7850	5.7 (5.2–6.2)
		66–85	89,644	10.1 (9.9–10.3)	6278	11.3 (10.5–12.1)

The age groups were chosen according to the three-stage model of Delanaye et al. [4]

The prevalence rates from model 2 with appliance of the chronicity criterion for the validation data sets are in the range of published prevalence rates when the chronicity criterion together with age-dependent lower reference limits of eGFR are applied to the study cohort (8.3/7.2/7.1% vs. 6%, [9, 10] and 8.3/7.2/7.1% vs. 5.1%, [16]) and lower than the estimated prevalence rates with one-time eGFR estimations without age-dependency in the definition of CKD (8.3/7.2/7.1% vs. 10–16% [9]).

## Discussion

Our findings confirm two important observations suggested by previous studies. First, the age-dependency of eGFR is remarkable with all three eGFR formulas. Second, using continuous age-dependent reference limits of eGFR together with the application of the chronicity criterion results in a remarkably lower CKD prevalence rate. Nevertheless, the results have to be discussed critically due to the retrospective study design.

Lower reference limits: When comparing the predicted lower reference limits from model 1 and model 2, higher reference limits in all three eGFR formulas are calculated with model 2. The underlying reason for this could be a share of patients with flares of acute kidney injury (e.g., deteriorations in fluid homeostasis with exsiccosis due to diuretics intake, impaired thirst, impaired renal function without proteinuria, leukocyturia, erythrocyturia and/or glucosuria, variable or impaired heart function) in the data sets for model 1, all of which may have an influence on the eGFR thresholds. By comparing the predicted lower reference limits to the ones from Delanaye’s work, as shown in Table 2, it becomes obvious that the reference limits from model 2 are still lower than the ones described by Delanaye and Pottel [4–6]. This may be explained by the methodology underlying the data collection. Although the 2.5th percentile was used as the lower limit in all three models (model 1, model 2 and Delanaye`s model), the collectives studied are likely to differ. A major limitation of the data underlying this study is that no further information on patients' health status was available due to retrospective analysis of data. Thus, information on comorbidities, such as hypertension or diabetes (no glucosuria at the time of measurement) or medication, that may affect renal function (e.g., RAS inhibitors, diuretics, or SGLT2 inhibitors) is missing and patients with stable but impaired eGFR may also be present in the data set despite inconspicuous urinalysis. This may result in a lower calculated 2.5th percentile than in the general population affecting the CKD prevalence calculated in this study. Another limitation may be that especially patients with advanced age may have been increasingly under medical surveillance due to a “reduced” kidney function and therefore healthy older individuals may be less likely to be entered into the data sets, which could lead to a higher prevalence of CKD in the cohort studied. Lastly, it should be remembered that patients with CKD but unstable renal function (e.g., additional AKI) were removed from the data set with the chosen study design. Thus, it must be concluded that the underlying data set is confounded by specific patient cohort analyzed by this laboratory and therefore is not fully representative of the general population. In addition, there are some minor overlaps of patients analyzed within the modeling and validation cohorts [e.g., for model 1 5662 patients from the modeling cohort were also included in the validation cohort (2.9% of the validation cohort)]. This overlap arises from the two data queries, which also overlapped in time to ensure a sufficiently high number of patients for reliable calculation of the models. The influence of this small proportion on the results of this study may be considered small, however has to be taken into account.

*CKD prevalence* It has been assumed that the CKD prevalence rate is higher when entering a one-time eGFR estimate compared to predictions with appliance of the KDIGO chronicity criterion [6, 9, 10]. Our results confirm this assumption (prevalence estimated from validation data set 1 vs. validation data set 2 as shown in Table 3 and 4). As a limitation, it must be noted that within the validation data sets, only the patients´ eGFR values were used to define CKD. Information about possible co-existing proteinuria was not available.

Furthermore, it could be shown that age-dependent lower reference limits based on a cohort with stable renal function lead to a higher CKD prevalence (prevalence estimated from model 1 vs. model 2 as shown in Table 3 and 4). This effect is most likely due to the higher 2.5th percentiles in model 2, since patients with stable kidney function were enrolled here, whereas patients with acute kidney injury were excluded from the data set.

Looking at the age-dependent prevalence rates, this study confirms that the prevalence of CKD increases with age. When results from Tables 3 and 4 are compared it is noticeable that for the age group 66–85 years a reversed pattern is present for the prevalence rates determined for validation data sets 1 and 2. While the age-independent prevalences were lower when using validation data set 2, higher prevalence rates were calculated in the 66–85 age group when using validation data set 2. Looking at the visualization of the models in Fig. 2, it is noticeable that for all three eGFR formulas, a lower eGFR drop-off is seen in model 2. This largely explains the discrepancy outlined above.

The strengths of this study are the size of the data sets, the measurement of creatinine as the basis for the eGFR calculation in the same laboratory and with the same methodology, the standardized and automated reading of the urine test strips for urinalysis, and the application of the chronicity criterion for estimation of the CKD prevalence rate. In addition, when using the 2.5th percentile as the definition of the lower limit of eGFR overdiagnosis of CKD in older patients is prevent. Furthermore, underdiagnosis of CKD in younger patients with an eGFR above 60 ml/min/1.73 m^2^ but below the age-specific 2.5th percentile of eGFR is precluded, as similarly shown in the work of Beghanem Gharbi et al. and Delanaye et al. [6, 16].

In summary, the findings set the basis for “real-life” prevalence rates of CKD when continuous age-dependent lower reference intervals of eGFR together with the chronicity criterion are applied. Further research should verify the established lower reference limits of model 2 in a carefully selected cohort in which the eGFR and other pathological conditions such as proteinuria are tested at the same time.
